# CBCT-based quantification of intrabony root volumes in adult natural teeth: An exploratory study

**DOI:** 10.4317/medoral.27700

**Published:** 2026-01-24

**Authors:** Jun-Sen Su, Wen Du, Ye-Ye Yang, Zeng-Zhao Wang, Shu Li, Ying-Ying Fan, Ye-Jun Cai, Xin Peng, Peng Ye

**Affiliations:** 1 Department of Stomatology, Jinan Stomatological Hospital, Jinan 250014, China; 2 Department of Oral and Maxillofacial Surgery, Peking University School and Hospital of Stomatology, Beijing 100081, China; 3 Department of Neurology, The Second Hospital, Cheeloo College of Medicine, Shandong University. Jinan 250033, China; 4 Department of Stomatology, Shandong Xiehe University (Guodian Campus), Jinan 250109, China; 5 Department of Stomatology, Beijing Hospital, National Center of Gerontology, Institute of Geriatric Medicine, Chinese Academy of Medical Sciences, Beijing 100730, China

## Abstract

**Background:**

Alveolar ridge preservation (ARP) is critical for implant dentistry outcomes, yet current graft material selection lacks standardized intrabony root volume benchmarks. This study aimed to establish baseline data for intrabony root volumes of adult teeth using CBCT.

**Material and Methods:**

This single-center cross-sectional study included 100 adults (50 males, 50 females) with complete natural dentition. CBCT-derived intrabony root volumes were measured for 10 target tooth positions using Mimics software. Volumetric data were analyzed for differences across tooth positions, jaw locations, and genders using parametric or nonparametric tests.

**Results:**

Intrabony root volumes varied by tooth position (first molar, first premolar, canine, lateral incisor, central incisor), with the largest volumes in upper first molars and smallest in lower central incisors. The intrabony root volumes of all target maxillary tooth positions were significantly higher than those of their mandibular homonymous counterparts (all P&lt;0.01). Males exhibited significantly larger intrabony root volumes than females across all tooth positions (P&lt;0.001).

**Conclusions:**

This exploratory study preliminarily establishes comprehensive reference data for adult intrabony root volumes across tooth positions, jaw locations, and genders, which may inform the development of tailored bone substitutes to address clinical limitations in graft volume estimation.

## Introduction

The preservation of the alveolar ridge after tooth extraction is paramount for predictable implant dentistry outcomes. Natural bone resorption inevitably compromises ridge dimensions, complicating subsequent prosthetic rehabilitation. Post-extraction, significant bone loss occurs within six months, characterized by substantial reductions in ridge width and height (1). Alveolar ridge preservation (ARP) techniques address this challenge by grafting biomaterials into extraction sockets, mitigating resorption and promoting new bone formation (2 - 5).

Despite the proven efficacy of ARP, current clinical practices suffer from a subjective and imprecise approach to determining graft material volumes, as standardized reference data for intrabony root volumes are nonexistent. Specifically, no studies have established quantitative benchmarks for root volume based on tooth position, arch location (maxillary vs. mandibular), or gender, limiting evidence-based graft selection. Consequently, clinicians often rely on empirical experience rather than volumetric measurements to estimate extraction socket size and guide graft material choices. Compounding this issue, commercial bone substitutes are available in limited size and mass specifications (6), leading to frequent overestimation of graft requirements and substantial resource wastage.

Fortunately, CBCT technology offers a viable solution for accurate 3D assessment of dental structures, enabling precise volumetric analysis of intrabony root sections. Multiple studies validate the feasibility of CBCT in segmenting and measuring root dimensions through automated or semi-automated image-processing techniques (7 - 10). These studies underscore CBCT's reliability as a non-invasive modality for obtaining quantitative 3D data, providing a solid foundation for objective root volume assessments in clinical research.

The present exploratory study aims to systematically quantify the intrabony root volume of adult natural teeth, rather than directly to validate its correlation with post-extraction socket volume. By examining a diverse cohort, we delineated variations in intrabony root volume across tooth positions, jaw locations and genders.

## Material and Methods

This single-center cross-sectional study was conducted in compliance with the Declaration of Helsinki, and its research protocol was reviewed and approved by the Medical Ethics Committee of Jinan Stomatological Hospital (JNSKQYY-2025-013). The study adheres to the Strengthening the Reporting of Observational Studies in Epidemiology (STROBE) guidelines, and written informed consent was obtained from all participants before data collection.

Participants

Eligible participants for our study were required to meet the following inclusion criteria: Adults 18 years old; complete natural permanent dentition; full-arch CBCT scans available. The exclusion criteria included: Diagnosed with chronic periodontitis; history of orthodontic treatment; congenital root anomalies; external root resorption.

Intrabony Root Volume Measurement

All CBCT images were acquired using a NEW-TOM (5G version FP) scanner with the following fixed parameters: Tube voltage 110kV, maximum tube current 32mA, slice thickness 0.3mm, and field of view 21cm×19cm.

CBCT images (anonymized) for each participant were imported into Mimics software 25.0 (Materialise, Leuven, Belgium). For tissue density reference, the CBCT system provided density values based on Hounsfield units (HU), which are noted to represent a local device-specific calibration scale rather than the standardized HU used in medical CT. These CBCT-derived HU values were used for relative density comparison within the same scan. In our study, the region-growing technique was applied for dentin segmentation with a threshold range of 1500~2000 Hounsfield Units (HU). This range not only aligns with dentin's intrinsic radiodensity but also effectively separates dentin from lower-density tissues (e.g., trabecular bone, typically&lt;1000 HU) while excluding high-density metallic artifacts (typically&gt;2500 HU) (11). Notably, all CBCT scans in this study were acquired using the same scanner device and fixed scanning parameters, eliminating inter-device variability.

**Figure 1:**
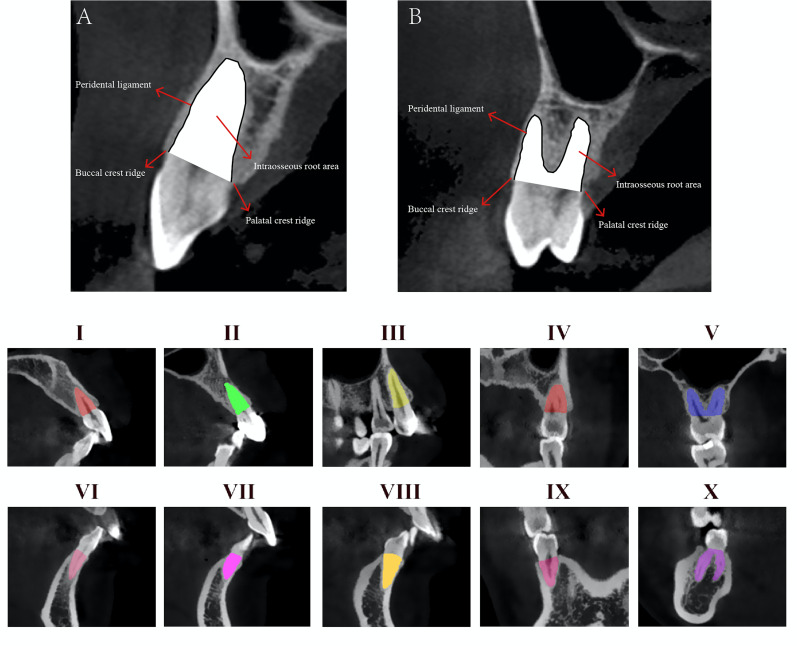
Panels A and B illustrate the delineation of the intrabony root area (white - shaded region) in a single-root tooth (A) and a multi-root tooth (B). For both tooth, the intrabony root area is anatomically defined by: A superior boundary formed by the horizontal line connecting the buccal crest ridge and palatal crest ridge; Peripheral boundaries corresponding to the interface between the periodontal ligament and the root surface. Panels I~X show representative variations of the intrabony root area (color-shaded regions) across diverse targeted tooth positions.

The intrabony root volume quantified in this study is specifically defined as the volume of the tooth root embedded in the alveolar bone and located inferior to the alveolar crest. The measurement protocol for this parameter followed a standardized, stepwise approach: Identification of the alveolar crest: First, the positions of the buccal and lingual alveolar crests were clearly delineated on CBCT images to establish the superior boundary of the intrabony root segment; cross-sectional area measurement: For each target tooth, cross-sectional slices were analyzed sequentially (from mesial to distal aspects). On each slice, the area of the root segment located below the predefined alveolar crest was manually outlined. The boundaries of this area were strictly defined as follows: The peripheral boundary was the periodontal ligament space, the superior boundary was the alveolar crests (Figure 1);

1

volumetric calculation: The intrabony root volume was ultimately computed using Mimics software. This software summed the manually delineated intrabony root areas across all consecutive slices and multiplied the total accumulated area by the CBCT slice thickness (0.3mm).

Finally, for data aggregation, volumes of left and right homologous teeth were averaged per jaw (maxilla and mandible) and the measurement data for each target tooth were derived from the mean values of two observers to ensure reliability.

The target teeth selected for intrabony root volume measurement included central incisors, lateral incisors, canines, first premolars, and first molars. For multi-root teeth, first premolars and first molars were included, as their predominantly divergent roots minimize accidental interradicular bone loss during extraction, avoiding discrepancies between pre-extraction intrabony root volume and post-extraction socket volume. All intrabony root volume measurements followed the standardized protocol mentioned earlier.

Ex vivo Validation of Intrabony Root Volume Measurement Protocol

To verify the accuracy of the CBCT-Mimics intrabony root volume measurement protocol, an ex vivo validation experiment was conducted prior to formal data collection.

**Figure 2:**
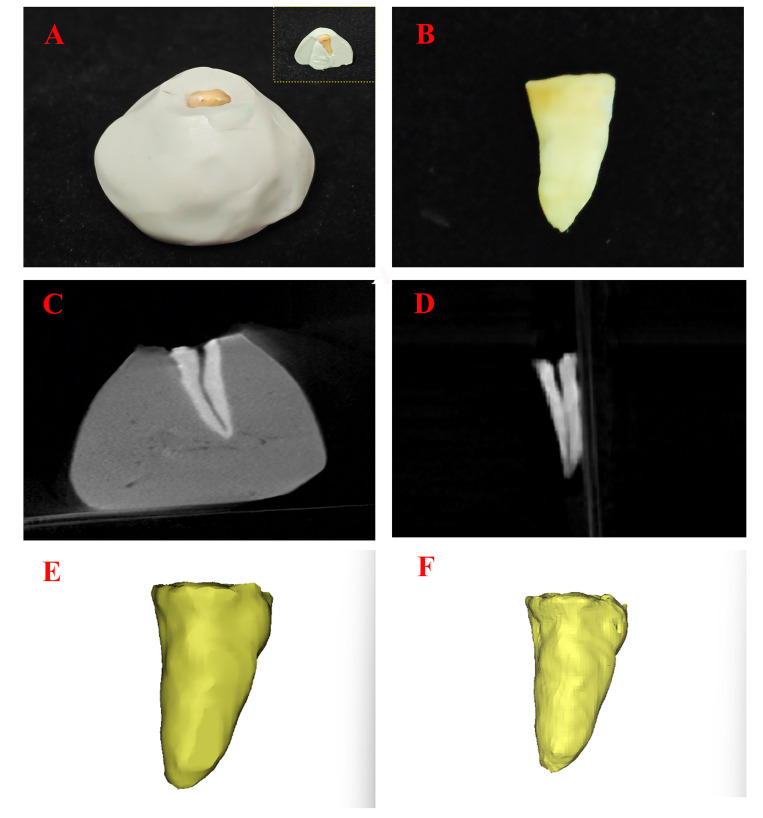
Schematic diagram of the ex vivo validation measurement protocol. A: Photomacrograph of the root segment embedded in silicone rubber (inset: Cross-sectional view of the embedded sample); B: Photomacrograph of the isolated root segment; C: CBCT cross-sectional image of the root segment within silicone rubber; D: CBCT cross-sectional image of the isolated root segment; E: Three-dimensional reconstruction of the simulated intrabony root (Mimics in vivo-simulated measurement); F: Three-dimensional reconstruction of the root (ex vivo direct measurement).

Ten extracted teeth with intact roots (2 of each first molar, first premolar, canine, lateral incisor, central incisor) underwent coronal resection to retain only the root segment. Two measurements were performed: Mimics in vivo-simulated measurement: Root segments were embedded in silicone rubber (alveolar bone mimicry), scanned with the same NEW-TOM 5G FP CBCT (110kV, 32mA, 0.3mm slice), and segmented to quantify simulated intrabony root volume; ex vivo direct measurement: Isolated roots were individually scanned, with pulp cavities filled via cavity-filling function before direct root volume quantification, and a schematic diagram of this measurement protocol is presented in Figure 2.

2

As Table 1 presents, the method achieved a mean absolute difference (MAD) of 5.37 mm³ (3.07~7.44mm³) and a mean relative error percentage (MRE%) of 2.36% (0.94%~4.65% for individual samples), confirming the protocol's excellent in vivo applicability and measurement accuracy.

Table 1

Study Power and Sampling

Sample Size Calculation

Sample size calculation was performed using PASS 21.0 (NCSS LLC, USA) to ensure statistical validity. Based on pilot data documenting a mean intrabony root volume of maxillary canines as 216.7±14.73mm³ (mean±SD), the following parameters were specified: Two-tailed alpha level () of 0.05, statistical power of 90% (=0.10), and a minimal clinically significant difference of 10mm³ (12). The parametric calculation yielded a minimum required sample size of N=92. To account for potential exclusion, the final sample size was adjusted to N=100, ensuring robust power and representativeness in the subsequent analysis.

Sampling Strategy

A consecutive sampling approach with gender stratification was implemented, and all eligible patient CBCT scans meeting inclusion criteria were consecutively enrolled. To ensure methodological rigor and analytical precision, this approach was further refined to achieve exact gender parity (N=50 per gender; 1:1 ratio) for balanced subgroup comparisons, mitigating confounding from gender-specific biological heterogeneity. This deliberate allocation strategy was designed to facilitate balanced subgroup comparisons by mitigating potential confounding variables inherent to gender-specific biological heterogeneity.

Measurement Bias Control

To mitigate measurement bias, a two-tier calibration strategy was employed. Two observers underwent rigorous training on standardized protocols for quantifying intrabony root volume using Mimics software. Inter-examiner and intra-examiner reliability were prospectively evaluated separately. The two examiners were cleared to independently measure the CBCT images of the 100 enrolled patients only after achieving an intraclass correlation coefficient (ICC) exceeding 0.90.

Patient identifying information was systematically anonymized prior to measurement. Each examiner conducted independent measurements of the entire dataset, and the final volume data of each target tooth for each patient was determined as the mean of the two assessments.

Statistical Analysis

All analyses were performed using SPSS 28.0 (IBM Corp., USA), with P&lt;0.05 denoting significance. With normality assessed via Shapiro-Wilk test, descriptive statistics are reported as: Mean±standard deviation (SD) and 95% confidence intervals (CI) of mean for normally distributed data; medians and Q1~Q3 (Q1: First quartile, Q3: Third quartile) for non-normally distributed data. Independent t test compared intrabony root volumes by gender, and paired t test evaluated intermaxillary differences; nonparametric Mann-Whitney U or Wilcoxon test were used for non-normal data. To control the family-wise error rate (FWER) caused by multiple statistical comparisons, Bonferroni correction was applied to two core sets of analyses: Paired comparisons of intrabony root volumes between maxillary and mandibular homonymous teeth; gender-based comparisons of intrabony root volumes across all 10 target tooth positions. The adjusted significance level (') was calculated as /n (where =0.05, n=number of comparisons) for each set of analyses.

## Results

Distribution of intrabony root volume by tooth position: Overall cohort analysis

As presented in Table 2, the Shapiro-Wilk test revealed that intrabony root volume data conformed to a normal distribution only for Lower central incisors (P=0.081); all other tooth positions showed non-normal distributions (P&lt;0.05). For mandibular central incisors, the volume was 95.98±19.50mm3, with a 95% CI of mean: 91.64~99.38mm3.

Table 2

For non-normally distributed data, results were reported as median and Q1~Q3: Upper first molar (442.94mm3, 369.36~527.19mm3); upper first premolar (211.15mm3, 188.86~273.94mm3); upper canine (304.95mm3, 241.22~357.97mm3); upper lateral incisor (165.74mm3, 129.78~190.34mm3); upper central incisor (191.57mm3, 154.93~223.22mm3); lower first molar (425.27mm3, 356.91~469.92mm3); lower first premolar (183.75mm3, 162.57~222.78mm3); lower canine (253.32mm3, 209.25~300.14mm3); and lower lateral incisor (130.66mm3, 109.16~144.41mm3).

Distribution of intrabony root volume by tooth position in the male cohort

In male cohort, for normally distributed data: The upper first premolar 258.95±62.17mm3, 95% CI of mean: 241.09~276.81mm3; the upper canine 346.62±76.27mm3, 95% CI of mean: 324.71~368.52mm3; the upper lateral incisor 178.82±42.79 mm3, 95% CI of mean: 166.53~191.11mm3; the upper central incisor 211.97±45.365 mm3, 95% CI of mean: 198.94~224.99mm3; lower canine 299.8±55.39mm3, 95% CI of mean: 284.07~315.56mm3; lower central incisor 101.70±19.08mm3, 95% CI of mean: 96.28~107.12mm3.

For non-normally distributed data: The upper first molar (501.19mm3, 426.02~550.48mm3); lower first molar (448.00mm3, 396.66~538.62mm3); lower first premolar (206.69mm3, 181.52~253.27mm3); lower lateral incisor (136.68mm3, 118.11~150.92mm3) (Table 3).

Table 3

Distribution of intrabony root volume by tooth position in the female cohort

In female cohort, for normally distributed data: The upper first molar 410.75±73.18mm3, 95% CI of mean: 389.95~431.54mm3; the upper central incisor 172.30±35.52mm3, 95% CI of mean: 162.20~182.39mm3; the lower first molar 393.63±74.98mm3, 95% CI of mean: 373.31~414.93mm3; the lower first premolar 169.39±29.97mm3, 95% CI of mean: 160.87~177.91mm3; lower central incisor 89.32±18.05mm3, 95% CI of mean: 84.19~94.44mm3.

For non-normally distributed data: The upper first premolar (202.77mm3, 172.92~228.80mm3); upper canine (267.88mm3, 225.97~307.34mm3); upper lateral incisor (145.83mm3, 123.46~177.07mm3); lower canine (212.09mm3, 186.64~240.99mm3); lower lateral incisor (117.40mm3, 104.72~135.850mm3) (Table 4).

Table 4

Comparison of intrabony root volumes between maxillary and mandibular homonymous teeth: Overall cohort analysis

Using the Wilcoxon signed-rank test for paired non-parametric analysis, we found that the intrabony root volumes of all target maxillary tooth positions (first molar, first premolar, canine, lateral incisor, central incisor) were significantly higher than those of their mandibular homonymous counterparts (all P&lt;0.01). After Bonferroni correction for 5 pairwise comparisons ('=0.05/5=0.01), all adjusted Pvalues remained &lt;0.01, confirming robust intermaxillary volumetric differences.

Gender-based comparison of intrabony root volume across tooth positions

Gender-based differences in intrabony root volume were assessed using independent t tests (for normally distributed data) or Mann-Whitney U tests (for non-normal data). Across all tooth positions, male intrabony root volume was significantly higher than that of females (all P&lt;0.001). After Bonferroni correction for 10 tooth position-specific comparisons ('=0.05/10=0.005), all adjusted Pvalues were &lt;0.005, indicating significant sexual dimorphism across all tooth positions.

## Discussion

This is the first study to systematically quantify intrabony root volume across various adult tooth positions using high-resolution CBCT-based volumetric analysis. To date, dental anatomy research has primarily focused on linear measurements of root length or diameter (13 - 15), with limited attention to three-dimensional volumetric characteristics of the intrabony root segment. Given the complete absence of prior literature on this specific volumetric parameter, our work fills a significant knowledge gap by establishing comprehensive baseline data for intrabony root volume across the main spectrum of adult dentition.

The intrabony root volume data from this study have substantial clinical relevance, particularly for ARP and subsequent implant rehabilitation. As the natural tooth's intrabony root segment forms the anatomical core dimension of the extraction socket, the inferred correlation between intrabony root volume and socket volume provides a precise reference for clinicians. Direct validation of this correlation in humans was not performed, as it would require immediate post-extraction CBCT scans in addition to pre-extraction imaging, an approach that would unnecessarily increase radiation exposure for patients and violate the ALARA (As Low As Reasonably Achievable) principle of radiation protection. Nevertheless, the baseline values reported herein (maxillary/mandibular central incisors, lateral incisors, canines, first premolars, first molars) still enable relatively accurate selection of bone graft substitutes during ARP procedures.

Notably, the selection of bone substitute volume in ARP remains heavily reliant on clinician experience, introducing substantial subjective variability, particularly among less experienced practitioners. This empirical approach often leads to imprecise graft volume estimation. Our findings address this critical gap by establishing objective, tooth position-specific volumetric benchmarks. Based on our results, the mean or median intrabony root volumes across different tooth positions are distributed around four primary ranges: 100mm³, 200mm³, 300mm³ and 400mm³. This suggests that newly developed bone substitute products could be designed and prefabricated in these four volume-based configurations. Clinicians can then use the tooth-specific volume data presented in this study to select the most closely matching bone substitute volume for alveolar ridge preservation following extraction of a particular tooth. For example, our data indicate a mean intrabony root volume of 307.65mm³ for an upper canine. In this case, clinicians may choose a bone substitute product with a volume of 0.3mL to achieve adequate socket filling. By focusing on volume (the critical factor determining accuracy of socket filling) this study provides clinically actionable guidance for evidence-based selection of bone graft material quantity, independent of the specific biomaterial used.

Moreover, a critical challenge in current ARP practice is the limited range of prefabricated bone substitute volumes available commercially (16 - 18), our comprehensive volumetric dataset addresses this limitation by defining precise, tooth-specific, gender-stratified benchmarks for intrabony root volume. These reference values provide a scientific foundation for the development of more tailored bone substitute products, ranging from incisors to molars.

Our study revealed a consistent pattern where intrabony root volumes were significantly larger in males than in females across all tooth positions. This sexual dimorphism carries direct implications for ARP, indicating that bone substitute volumes should be adjusted according to gender even for the same tooth position, with higher volumes required for male patients. The observed gender differences align with well-documented anatomical variations in the human dentition and jaws. Previous studies have established that males typically exhibit larger overall jaw dimensions, including increased alveolar bone volume and thicker cortical plates, compared to females (19 - 20). Additionally, tooth size dimorphism, with males displaying larger crown and root dimensions across multiple tooth positions, has been consistently reported in dental anthropology and anatomy research (21 - 23). These broader skeletal and dental developmental differences likely underpin the volumetric disparities in intrabony root segments observed in our study.

In our study, the exclusively Han Chinese cohort may limit the generalizability of the findings to more diverse populations, as tooth anatomical structures are known to vary across ethnic groups. For example, a systematic review reported that maxillary central incisors in Caucasians exhibit greater crown width and length compared to those in Asians, along with a lower width-to-length ratio (24). Additionally, Kumar et al. observed that Tamil population maxillary canines had shorter root length and reduced mesiodistal width relative to conventional norms, with distinct root-crown proportions compared to other non-Western groups (25). These ethnically related morphological differences may contribute to variations in root volume.

This study has several limitations. First, as a single-center investigation, the geographically restricted sample may introduce selection bias, limiting the generalizability of the volumetric benchmarks to broader clinical populations. Second, second molars and second premolars were excluded from volumetric analysis, which might overlook subtle morphological and volumetric differences in these teeth and introduce methodological bias. Third, the 0.3mm CBCT slice thickness may cause partial volume effects that blur structural interfaces; this was mitigated by cross-referencing multiplanar slices for segmentation. While sufficient for quantifying macroscopic intrabony root volume, this slice thickness cannot resolve fine anatomical structures. A further limitation is the unvalidated assumption that intrabony root volume approximates post-extraction alveolar socket volume. The primary source of potential discrepancy is the periodontal ligament (PDL). While this unverified link does not diminish the dataset's clinical value, we plan to validate this correlation via future animal studies using paired pre-extraction and immediate post-extraction CBCT imaging of the same subjects.

## Conclusions

This exploratory study preliminarily establishes comprehensive reference data for adult intrabony root volumes across tooth positions, jaw locations, and genders. Furthermore, our findings may inform the development of tailored bone substitutes, toward addressing current clinical limitations in graft volume estimation.

## Figures and Tables

**Table 1:** Table Ex vivo validation of intrabony root volume measurement.

Number	Tooth position	Mimic in vivo intrabony root volume (mm3)	Ex vivo intrabony root volume (mm3)	AD (mm3)	MAD (mm3)	RE%	MRE%
1	First molar	407.22	401.95	5.27	5.37	1.31	2.36
2	First Molar	414.62	409.7	4.92		1.2	
3	First premolar	257.25	249.81	7.44		2.98	
4	First premolar	216.8	211.78	5.02		2.37	
5	Canine	290.73	295.06	4.33		1.47	
6	Canine	328.17	325.1	3.07		0.94	
7	Lateral incisor	162.55	155.32	7.23		4.65	
8	Lateral incisor	159.3	164.28	4.98		3.03	
9	Central incisor	210.7	216.59	5.89		2.72	
10	Central incisor	194.43	188.9	5.53		2.93	

AD: Absolute difference (|Mimic in vivo value-Ex vivo value|); MAD: Mean absolute difference; RE%: Relative error percentage (Mimic in vivo value-Ex vivo value/Ex vivo value)x100%; MRE%: Mean relative error percentage.

**Table 2:** Table Distribution of intrabony root volume by tooth position in overall cohort.

Jaw position	Tooth position	Shapiro-Wilk test	Mean (mm3)	SD (mm3)	95%CI of mean (mm3)	Median (mm3)	Q1~Q3 (mm3)
Maxilla	First molar	P<0.01				442.94	369.36~527.19
	First premolar	P<0.01				211.15	188.86~273.94
	Canine	P<0.01				304.95	241.22~357.97
	Lateral incisor	P<0.01				165.74	129.78~190.34
	Central incisor	P=0.02				191.57	154.93~223.22
Mandible	First molar	P<0.01				425.27	356.91~469.92
	First premolar	P<0.01				183.75	162.57~222.78
	Canine	P<0.01				253.32	209.25~300.14
	Lateral incisor	P<0.01				130.66	109.16~144.41
	Central incisor	P=0.08	95.98	19.50	91.64~99.38		

Sample size N=100; data normality assessed via Shapiro-Wilk test; descriptive statistics were reported based on data distribution: Mean±SD and 95%CI of mean for normally distributed data, median and Q1~Q3 for non-normally distributed data; unit: mm³.

**Table 3:** Table Distribution of intrabony root volume by tooth position in male cohort.

Jaw position	Tooth position	Shapiro-Wilk test	Mean (mm3)	SD (mm3)	95%CI of mean (mm3)	Median (mm3)	Q1~Q3 (mm3)
Maxilla	First molar	P<0.01				501.19	426.02~550.48
	First premolar	P=0.08	258.95	62.17	241.09~276.81		
	Canine	P=0.46	346.62	76.27	324.71~368.52		
	Lateral incisor	P=0.31	178.82	42.79	166.53~191.11		
	Central incisor	P=0.53	211.97	45.36	198.94~224.99		
Mandible	First molar	P<0.01				448.00	396.66~538.62
	First premolar	P<0.01				206.69	181.52~253.27
	Canine	P=0.10	299.81	55.39	284.07~315.56		
	Lateral incisor	P<0.01				136.68	118.11~150.92
	Central incisor	P=0.40	101.70	19.08	96.28~107.12		

3

**Table 4:** Table Distribution of intrabony root volume by tooth position in female cohort.

Jaw position	Tooth position	Shapiro-Wilk test	Mean (mm3)	SD (mm3)	95%CI of mean (mm3)	Median (mm3)	Q1~Q3 (mm3)
Maxilla	First molar	P=0.60	410.75	73.18	389.95~431.54		
	First premolar	P=0.01				202.77	172.92~228.80
	Canine	P<0.01				267.88	225.97~307.34
	Lateral incisor	P=0.02				145.83	123.46~177.07
	Central incisor	P=0.06	172.30	35.52	162.20~182.39		
Mandible	First molar	P=0.16	393.63	74.98	372.31~414.93		
	First premolar	P=0.06	169.39	29.97	160.87~177.91		
	Canine	P<0.01				212.09	186.64~240.99
	Lateral incisor	P=0.02				117.40	104.72~135.85
	Central incisor	P=0.19	89.32	18.05	84.19~94.44		

4

## Data Availability

All data supporting the findings of this study are available on request from the Correspondence.

## References

[1] Tan WL, Wong TL, Wong MC, Lang NP (2012). A systematic review of post-extractional alveolar hard and soft tissue dimensional changes in humans. Clin Oral Implants Res.

[2] Gong H, Zhao Y, Chen Q, Wang Y, Zhao H, Zhong J (2022). 3D bio-printing of photocrosslinked anatomically tooth-shaped scaffolds for alveolar ridge preservation after tooth extraction. J Mater Chem B.

[4] Quirynen M, Siawasch S, Temmerman A, Cortellini S, Dhondt R, Teughels W (2023). Do autologous platelet concentrates (APCs) have a role in intra-oral bone regeneration? A critical review of clinical guidelines on decision-making process. Periodontol 2000.

[5] Avila-Ortiz G, Elangovan S, Kramer KW, Blanchette D, Dawson DV (2014). Effect of alveolar ridge preservation after tooth extraction: A systematic review and meta-analysis. J Dent Res.

[6] Hikita A, Chung UI, Hoshi K, Takato T (2017). Bone regenerative medicine in oral and maxillofacial region using a three-dimensional printer. Tissue Eng Part A.

[7] MacDonald D, Telyakova V (2024). An overview of cone-beam computed tomography and dental panoramic radiography in dentistry in the community. Tomography.

[8] Lin J, Zheng Q, Wu Y, Zhou M, Chen J, Wang X (2025). Quantitative analysis and clinical determinants of orthodontically induced root resorption using automated tooth segmentation from CBCT imaging. BMC Oral Health.

[9] Zhang Y, Liu Y, Liu T, Zhang J, Lin P, Liu D (2024). Evaluation of CBCT reconstructed tooth models at different thresholds and voxels and their accuracy in fusion with IOS data: An in vitro validation study. BMC Oral Health.

[10] Liu W, Shao J, Li S, Al-Balaa M, Xia L, Li H (2021). Volumetric cone-beam computed tomography evaluation and risk factor analysis of external apical root resorption with clear aligner therapy. Angle Orthod.

[11] Tan M, Cui Z, Zhong T, Fang Y, Zhang Y, Shen D (2024). A progressive framework for tooth and substructure segmentation from cone-beam CT images. Comput Biol Med.

[12] Ozdede M, Akay G, Karadag Atas O (2025). Influence of CBCT device, voxel size, and segmentation software on the accuracy of tooth volume measurements. BMC Oral Health.

[13] Ruetters M, Gehrig H, Awounvo S, Kim TS, Doll S, Alexandrou K (2024). Tooth segmentation by low-dose CBCT for orthodontic treatment planning: Explorative ex vivo validation. J Orofac Orthop.

[14] Abulhamael AM, Barayan M, Makki LM, Alsharyoufi SM, Zahran S (2024). The accuracy of cone beam computed tomography scans in determining the working length in teeth requiring non-surgical endodontic treatment: A retrospective clinical study. Cureus.

[15] Izadi A, Golmakani F, Kazeminejad E, Mahdavi Asl A (2024). Accuracy of working length measurement using cone beam computed tomography at three field of view settings, conventional radiography, and electronic apex locator: An ex-vivo study. Eur Endod J.

[16] Ferraz MP (2023). Bone grafts in dental medicine: An overview of autografts, allografts and synthetic materials. Materials (Basel).

[17] Misztal-Kunecka A, Prządka P, Jeż M, Dzimira S (2024). Determining hydroxyapatite filling volume for the treatment of post-extraction alveoli based on measurements of alveolar volume in relation to the body weight of dogs. Vet Sci.

[18] Wang J, Qi X, Zhou Y, Wang G, Yang Y, Jiang T (2023). Stabilization of Bio-Oss® particulates using photocurable hydrogel to enhance bone regeneration by regulating macrophage polarization. Front Bioeng Biotechnol.

[19] Tokpınar A, Alkan Y (2025). The role of mandibular morphological markers in determining sex and age: Anatomical and atropometric analysis. Folia Morphol (Warsz).

[20] Matschke J, Farahzadi S, Sembdner P, Holtzhausen S, Kroschwald L, Korn P (2024). A cross-sectional study of the anatomy of the jaws of a central-European caucasian population using cone beam computer tomography as a prerequisite for designing pre-formed calcium phosphate cement scaffolds. Ann Anat.

[21] de Bruin SG, Ishwarkumar-Govender S, Pillay P (2024). Anatomical facial characteristics of teeth and tooth analysis. Dent J (Basel).

[22] Cao R, Qiu P, Ni J, Xu H, Pan H, Cao Y (2023). A comprehensive analysis of clinical crowns in young of han nationality with normal occlusion using intraoral scanning. Int J Clin Pract.

[23] Prabhu N, Issrani R, Rao K, Saleh Albalawi A, Mahali Alharbi B, Noman Alanazi AW (2024). Analysis of gender dimorphism and assessment of racial variation through odontometric technique: A cross-sectional study. Cureus.

[24] Wang Y, Song Y, Zhong Q, Xu C (2021). Evaluation of influence factors on the width, length, and width to length ratio of the maxillary central incisor: A systematic review and meta-analysis. J Esthet Restor Dent.

[25] Kumar SM, Pandiar D, Poothakulath Krishnan R, Ramadoss R (2024). Estimation of tooth dimensions and golden divine ratio in extracted human permanent maxillary and mandibular canines in a cohort of tamil ethnicity. Cureus.

